# Drivers of bacterial diversity dynamics in permeable carbonate and silicate coral reef sands from the Red Sea

**DOI:** 10.1111/j.1462-2920.2011.02494.x

**Published:** 2011-07

**Authors:** Sandra Schöttner, Barbara Pfitzner, Stefanie Grünke, Mohammed Rasheed, Christian Wild, Alban Ramette

**Affiliations:** 1 Microbial Habitat Group, Max Planck Institute for Marine MicrobiologyCelsiusstrasse 1, 28359 Bremen, Germany; 2 Coral Reef Ecology Group (CORE), Leibniz Center for Tropical Marine EcologyFahrenheitstrasse 6, 28359 Bremen, Germany; 3 Marine Science Station, Jordan University and Yarmouk UniversityPO Box 195, 77110 Aqaba, Jordan

## Abstract

Permeable sediments and associated microbial communities play a fundamental role in nutrient recycling within coral reef ecosystems by ensuring high levels of primary production in oligotrophic environments. A previous study on organic matter degradation within biogenic carbonate and terrigenous silicate reef sands in the Red Sea suggested that observed sand-specific differences in microbial activity could be caused by variations in microbial biomass and diversity. Here, we tested this hypothesis by comparing bacterial abundance and community structure in both sand types, and by further exploring the structuring effects of time (season) and space (sediment depth, in/out-reef). Changes in bacterial community structure, as determined via automated ribosomal intergenic spacer analysis (ARISA), were primarily driven by sand mineralogy at specific seasons, sediment depths and reef locations. By coupling ARISA with 16S-ITS rRNA sequencing, we detected significant community shifts already at the bacterial class level, with *Proteobacteria* (*Gamma*-, *Delta*-, *Alpha*-) and *Actinobacteria* being prominent members of the highly diverse communities. Overall, our findings suggest that reef sand-associated bacterial communities vary substantially with sand type. Especially in synergy with environmental variation over time and space, mineralogical differences seem to play a central role in maintaining high levels of bacterial community heterogeneity. The local co-occurrence of carbonate and silicate sands may thus significantly increase the availability of microbial niches within a single coral reef ecosystem.

## Introduction

Tropical coral reefs are highly diverse and productive ecosystems, where complex (a)biotic environmental gradients provide multiple habitats and niches over time and space (Bellwood *et al*., 2004; Ainsworth *et al*., 2010). The unseen majority of reef organisms comprise microbial communities which, due to their versatile metabolic capacities, are involved in numerous pelagic and benthic processes (e.g. Capone *et al*., 1992; Sakka *et al*., 2002; Werner *et al*., 2008). Especially heterotrophic bacteria inhabiting the unconsolidated reef frameworks and sandy sediments are recognized as key players in the remineralization of organic matter directly within the reef system (Wild *et al*., 2004; Alongi *et al*., 2007). The concomitant recycling of nutrients is particularly crucial for coral reefs to maintain high levels of primary production and biomass in extremely oligotrophic seawater (D'Elia and Wiebe, 1990; Tribble *et al*., 1994).

Sandy reef sediments are highly permeable structures, where current-, wave- and tide-induced pressure gradients promote advective transport between sediment porewater and the overlying water column (Wheatcraft and Buddemeier, 1981; Huettel *et al*., 2003). By retaining dissolved and suspended matter that is hydraulically flushed into the (upper layers of the) sediment matrix and subsequently metabolized by grain-associated bacteria, these sands function as biocatalytic filter systems that ensure efficient pelagic-benthic coupling (Falter and Sansone, 2000; Wild *et al*., 2004).

Biogenic carbonate sands, which are mainly composed of fragmentary remains from calcifying organisms, usually represent the dominant sediment type in coral reef environments. Depending on local atmospheric andgeological events, terrigenic silicate sands may also occur, especially in fringing reefs that receive terrestrial deposits from nearby river mouths (Reiss and Hottinger, 1984). Carbonate and silicate particles substantially differ in their physico-chemical properties, such as surface structure, dissolution kinetics, light and heat attenuation, or buffering capacity (Schroeder and Purser, 1986), and their sediment matrices show clear differences in grain size distribution and sorting. In general, carbonate sands are characterized by a higher permeability, porosity and specific surface area than silicate sands, mainly due to the relatively large grain size and highly porous grain structure of the carbonate fraction (Rasheed *et al*., 2003a; Al-Rousan *et al*., 2006; Wild *et al*., 2006). By implying specific abiotic and biotic conditions within each sediment matrix, mineralogical composition may therefore represent a proxy parameter that informs on niche creation and hence diversification of associated microbial communities.

In a fringing reef in the Northern Red Sea, permeable carbonate and silicate sands co-occur within the same reef system. Although being exposed to identical environmental conditions, both kinds of sediments exhibit strong divergence in their spatio-temporal nutrient dynamics, as well as in their organic matter filtration and degradation capacities (Rasheed *et al*., 2003a,b; Wild *et al*., 2005). For example, the addition of energy-rich natural particulate organic matter stimulates the local sedimentary oxygen consumption significantly more in the carbonate than in the nearby silicate sands (Wild *et al*., 2005).

In this study, we specifically tested whether the contrasting mineralogy of those co-occurring carbonate and silicate reef sands would have marked effects on bacterial abundance and community structure. Given the strong spatio-temporal variations in nutrient and organic matter concentration prevailing in these sands, we also assessed seasonal and spatial effects as covariables. In addition, we identified the main bacterial taxa colonizing both sand types and described their changes in time and space by coupling community fingerprinting (i.e. ARISA) profiles with the corresponding 16S rRNA sequence information.

## Results and discussion

Based on sedimentological analyses, the two different types of permeable sediments from a fringing reef of the northern Gulf of Aqaba were clearly classified as coarsely grained carbonate and medium-grained silicate sands. For sand-associated bacteria, those mineralogical differences likely determine the overall mode and extent of colonization and niche differentiation, causing clear variations in cell abundance, distribution and overall community structure. In particular, grain texture and micro-topography are fundamentally important parameters controlling bacterial distribution and diversity in sediments (Meadows and Anderson, 1966; Weise and Rheinheimer, 1978). While the comparatively round silicate particles exhibit a rather regular, smooth surface, carbonate particles feature many micro-discontinuities, such as crevices, pores and depressions (Wild *et al*., 2006), which give each grain a highly porous and heterogeneous character and thus a larger specific surface area. Such increased supply of interfaces and micro-gradients promotes substrate availability, shelter from mechanical damage or predation (Frankel, 1977; DeFlaun and Mayer, 1983; Meyer-Reil, 1994, and references therein) as well as the formation of extracellular polysaccharides that serve as multi-purpose binding agents in cell attachment and biofilm formation (Meyer-Reil, 1994 and references therein).

### Changes in microbial cell number

With 3.1 ± 0.9 × 10^9^ and 1.5 ± 0.5 × 10^9^ cm^−3^ microbial cells in carbonate and silicate sands, respectively, no significant difference in cell number was observed (Student's *t*-test, *P* > 0.05). Overall, these estimates agree well with those for carbonate sediments of the Great Barrier Reef (Hansen *et al*., 1987) and two Hawaiian reefs (Sørensen *et al*., 2007; Rusch *et al*., 2009). However, microbial counts turned out to be considerably higher than previously determined cell numbers in the same area (Rasheed *et al*., 2003b). This may be partly attributed to methodological differences, as we used a refined version of the AODC method (Wild *et al*., 2006), which considers the fact that cells are also trapped within the highly porous sediment matrix.

The coarse-grained fraction of permeable sands can be expected to hold the major share of benthic bacteria (Nickels *et al*., 1981; Rusch *et al*., 2003), reaching levels of 10^9^–10^10^ cells cm^−3^, with up to one order of magnitude higher cell numbers in carbonate compared with silicate sands (Wild *et al*., 2005; Rusch *et al*., 2006). In our study, similar levels of microbial biomass were obtained for both sand types, despite marked differences in grain complexity. This may be due to the specific surface area of single carbonate particles being eventually down-balanced by their relatively high grain size. The total surface area effectively available for bacterial colonization in the whole carbonate sediment matrix would thus be reduced to a level similar to that offered by the smaller-grained silicate sediment.

### Variation in ARISA-derived OTU_A_ numbers

**Fig. 1:**
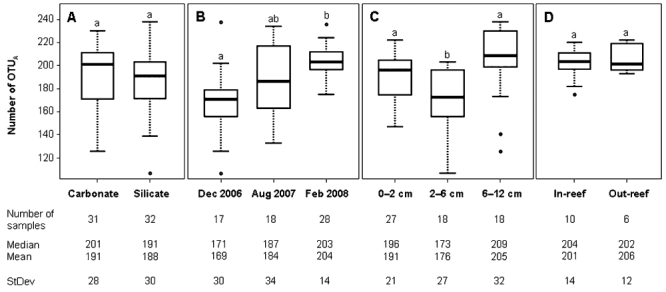
Number of ARISA-derived OTU_A_ per (A) sand type, (B) season, (C) sediment depth and (D) in/out-reef location. Top, middle and bottom lines of boxes represent the 25th (lower hinge), 50th (median) and 75th (upper hinge) percentiles; whiskers represent the non-extreme sample minimum and maximum (i.e. less than 1.5 × the inter-quartile range of the box); outliers above and below whiskers denote extreme values. Different letters above each box denote a significant mean difference in OTU_A_ number based on pairwise Wilcoxon–Mann–Whitney tests. The additional information specifies the total number of samples accounted for (including replicates), as well as the corresponding median, mean and standard deviation.

OTU_A_ numbers (i.e. the total of binned ARISA peaks) per sample ranged from 163 to 226 out of 423 OTU_A_ for the whole data set, and were similar to numbers reported by other ARISA-based studies on reef sands (Hewson and Fuhrman, 2006), different coastal marine sediments (Danovaro and Pusceddu, 2007; Hewson *et al*., 2007; Böer *et al*., 2009), or the water column at the target reef (S. Schöttner, C. Wild, A. Boetius and A. Ramette, unpubl. data). When averaged over sampling time, sediment depth, and in-/out-reef location, carbonate and silicate sands contained a similar mean total of 191 and 188 OTU_A_ respectively [Kruskal–Wallis test (KW), *P* = 0.56; Fig. 1A]. Nevertheless, a seasonal trend was clearly evidenced (KW, *P* < 0.001; Fig. 1B), with lowest and highest OTU_A_ numbers in December 2006 (169 OTU_A_) and February 2008 (204 OTU_A_) respectively. When considering each sand type individually, the temporal effect appeared to be mainly observed in carbonate (KW, *P* < 0.01), but not in silicate samples (KW, *P* = 0.09; data not shown). In their ARISA-based study on intertidal sand communities of the North Atlantic, Böer and colleagues (2009) also found the lowest OTU_A_ numbers in fall (November), but the highest numbers in summer (August) instead of winter (February). This may be explained by general, ecosystem-specific differences in seasonal dynamics between virtually permanently submerged tropical reefs and strongly tide-affected temperate sand flats, which essentially include temporal shifts in peaks of allochthonous nutrient concentrations and ensuing primary production. In addition, OTU_A_ numbers exhibited sediment depth-related differences (KW, *P* < 0.001; Fig. 1C), with a slight mean decrease from the surface (191 OTU_A_) to the middle layer (176 OTU_A_) and a subsequent increase to the deeper layer (205 OTU_A_). Yet again, the two sand types revealed substantial difference, as this vertical effect proved significant only for the silicate (KW, *P* < 0.001), but not for the carbonate communities (KW, *P* = 0.07; data not shown). Vertical variations in ARISA-derived OTU_A_ number were also identified in Australian reef sediments (Hewson and Fuhrman, 2006), with a clear subsurface maximum and subsequent OTU_A_ decrease between 3–5 cm sediment depth. No marked horizontal differences in OTU_A_ number were detected in out-reef versus in-reef surface sands (KW, *P* = 0.91; Fig. 1D).

### Patterns of community change between samples

Shifts in OTU_A_ presence–absence revealed that many OTU_A_ were present in all samples, with generally high numbers of OTU_A_ shared between the different sand types, seasons and sediment depths, as well as between in- and out-reef sites. Overall 377 (out of 423) OTU_A_ were detected in both sand types together, irrespective of season or sediment depth. With 22 and 24 OTU_A_ being unique to carbonate and silicate samples, respectively, the two sands thereby exhibited a general OTU_A_ overlap of about 95%. At defined seasons and sediment depths, however, it decreased to 52–73% (see Fig. S1). While only 4 and 3 OTU_A_ turned out as specific to December 2006 and August 2007, respectively, 32 OTU_A_ were associated with February 2008, amounting to an overall seasonal OTU_A_ overlap of 93–99%. When studying OTU_A_ presence per sediment depth, 20 OTU_A_ were found only in the surface layer, 2 OTU_A_ in the middle layer, and 8 OTU_A_ in the deep layer, corresponding to a vertical OTU_A_ overlap of 95–99%. At the sediment surface (total pool of 382 OTU_A_), in- and out-reef sediment samples contained 50 and 35 unique OTU_A_, respectively, resulting in an OTU_A_ overlap with location of about 87–91%.

**Fig. 2:**
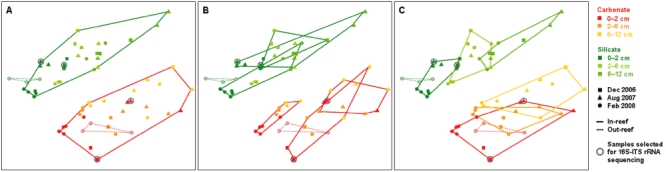
NMDS ordination (Bray–Curtis distance) of ARISA-derived bacterial community profiles, with at least three sediment replicates per sample. *A posteriori* groupings are specified according to (A) sand type, (B) season and (C) sediment depth, all including in/out-reef location. Samples chosen for 16S-ITS rRNA clone libraries are encircled. Objects plotting closer to each other share a more similar community structure relative to other, more distant objects. The low stress value of 0.18 indicates the goodness-of-fit of the two-dimensional representation compared with the original matrix.

When bacterial community patterns in carbonate and silicate sands were visualized by NMDS (Fig. 2A–C), the sand type had the largest structuring effect (Fig. 2A), followed by the effects of sampling time, sediment depth and sampling location (Fig. 2B and C). Those group separations were also supported by significant anosim results, where sand type was associated with the highest community separation (anosim*R* = 0.56, *P* < 0.001). Seasonal and vertical community differences were also significant (anosim*R* = 0.44 and *R* = 0.27 respectively; both *P* < 0.001) and greatly mineralogy-dependent. Within carbonate sands, for example, samples were mainly separated by season (anosim*R* = 0.80, *P* < 0.001), with a noticeable segregation of winter (February 2008) from fall (December 2006) and summer (August 2007) communities (Fig. 2B). Sediment depth-related patterns did not appear very pronounced (anosim*R* = 0.12, *P* < 0.05), but were consistent with a gradual change in community structure from the surface down to the deep layer (Fig. 2C). Variation in the silicate sands, by contrast, largely followed a vertical pattern (anosim*R* = 0.59, *P* < 0.001; Fig. 2C), while a seasonal effect, albeit significant (anosim*R* = 0.49, *P* < 0.001), was not clearly observed in the NMDS ordination (Fig. 2B). Cluster analysis results largely confirmed all major community groupings revealed by NMDS (data not shown).

As sand type, season and sediment depth significantly explained changes in community structure, canonical RDA variation partitioning was used to disentangle the respective effects of every factor while taking the other ones into account. Each factor specifically contributed with 14% (sand type), 8% (season) and 8% (sediment depth) of the total community variation (Table 1), with only 1% of co-variation between season and sediment depth (data not shown). While sand type represented the dominant factor the relative effects of season and sediment depth differed considerably when carbonate and silicate samples were examined separately (Table 1). In carbonate sands alone, season (14%) exerted an almost threefold higher influence on the bacterial community structure than sediment depth (5%). Conversely, changes characterizing only the silicate sand communities were more due to sediment depth (19%) than to season (12%). Those observations were also supported by an analysis of factor interactions (Table 1), which indicated that significant structuring effects were also due to the combinations of sand type, season and sediment depth. For instance, the sum of the pure effects of sand type and season in the model accounted for 22% of the explained variation, while the inclusion of their crossed effect increased the explained community changes to 25% (Table 1). A similar relation was found for the combined effect of sand type and sediment depth, where the explained variation increased from 22% to 24%. Hence, season and sediment depth not only provided additional structuring for an already mineralogy-controlled community variation, but their respective effect depended directly on the prevailing sand type.

**Table 1:** Canonical partitioning of the bacterial variation into the relative effects of sand type, season and sediment depth, individually or in combination

Multivariate model	d.f.	*R*^2^_adj_	*F*
Carbonate and silicate			
Type + Season + Depth	3	0.30	8.812***
Type | (Season, Depth)	1	0.14	11.342***
Season | (Type, Depth)	1	0.08	2.729***
Depth | (Type, Season)	1	0.08	6.979***
Type × Season	3	0.25	7.187***
Season × Depth	3	0.17	4.929***
Depth × Type	3	0.24	6.775***
Carbonate			
Season + Depth	2	0.21	4.590***
Season | Depth	1	0.14	5.705***
Depth | Season	1	0.05	2.658***
Season × Depth	3	0.24	3.888***
Silicate			
Season + Depth	2	0.31	7.227***
Season | Depth	1	0.12	5.780***
Depth | Season	1	0.19	8.339***
Season × Depth	3	0.33	5.677***

For each global or partial model, the number of degrees of freedom (d.f.), amount of explained variation (*R*^2^_adj_), *F* statistic and corresponding significance levels (^***^ *P* < 0.001) as assessed by 999 permutations are indicated. Symbols (+, ×) denote models with additive or interaction effects, respectively, while a vertical bar ( | ) indicates a partial regression model, wherein the effects of the factors on the right-hand side of the bar were controlled for while assessing main effects.

Overall, the different seasons clearly featured temporally distinct bacterial assemblages, with the winter (February 2008) samples showing the strongest segregation in community structure and high homogeneity in beta diversity. It is assumed that this relates to the overall enhanced primary productivity during fall and winter (November–March; Levanon-Spanier *et al*., 1979), which is triggered by the wind-driven inflow of Red-Sea offshore water into the Gulf of Aqaba during October, and concomitant upwelling of deep, nutrient-rich water to the surface and into the reef (Al-Najjar, 2000; Manasrah *et al*., 2006). This period ends in May, when the intrusion of offshore water decreases and nutrient concentrations in the reef drop and stabilize again. According to the organic carbon and nutrient inventories measured by Rasheed and colleagues (2003b) in the coastal water, those transitions (which are clearly marked by changes in, e.g. wind speed/direction, temperature or salinity) generate two main seasonal patterns: a winter period (with increased substrate concentrations) that includes October to April, and a summer period (with decreased substrate concentrations) that lasts from July until September. Due to effective pelagic-benthic coupling, whereby advective fluid exchange transmits seasonal changes from the water column into the porewater, carbon and nutrient inventories of the surface sediment layer clearly mirror the changes in the water column (Rasheed *et al*., 2003b). This not only supports the existence of a nutritional link between seasonal dynamics and sediment-associated communities, but also validates our finding of a stronger seasonal imprint on bacterial assemblages inhabiting the highly advection-driven carbonate as compared with the mostly diffusion-limited silicate sands.

In contrast to seasonal trends, vertical differences in community structure, albeit unequally pronounced in carbonate and silicate sands, are likely related to gradients in redox potential as well as organic matter and nutrient concentration. Stratification of the sediment column generally features steep transitions between the well-oxygenated, substrate-rich surface layer and the underlying suboxic and anoxic deeper layers, which is why both sands exhibited community variations from 0 cm down to 12 cm. However, while an enhanced advective flushing of the carbonate matrix results in rapidly changing porewater geochemistry (Rusch *et al*., 2009) with intensified and deeper-reaching supply of oxygen, organic matter and nutrients (Rasheed *et al*., 2003b), the comparatively reduced transport in silicate sands likely causes a much higher stratification of particles than in carbonate sands. In addition to these physico-chemical constraints, macrofaunal activity such as bioturbation (Riddle, 1988), grazing (Moriarty *et al*., 1985; Epstein, 1997) or nutrient regeneration (Uthicke, 2001) can also play a substantial role in structuring bacterial communities associated with the sediment surface layer.

Altogether, not only sand type-specific changes *per se*, but also seasonal and vertical shifts in bacterial diversity clearly reflected the fundamental difference in mineralogy and, ultimately, filtration efficiency of carbonate and silicate reef sands. Apart from the significant temporal and spatial imprints detected in all samples, carbonate communities shifted mainly with season while silicate communities rather shifted vertically with sediment depth.

### 16S-ITS rRNA clone library analyses

The four clone libraries (Ca06/Ca07, Si06/Si07) constructed from carbonate and silicate surface sands sampled in December 2006 and August 2007 yielded a total of 283 non-chimeric sequences (average length of the 16S rRNA gene: 1050 bp). Those corresponded to 168 OTU_S_ (≥ 98% identity), with the highest and lowest OTU_S_ richness found in library Ca06 (66 OTU_S_) and library Si06 (38 OTU_S_) respectively (Table 2). Total richness estimates (Chao1) showed that both fall libraries (Ca06 and Si06) contained the highest possible number of OTU_s_ (Table 2), with the carbonate sample being even more diverse than the silicate sample. Rarefaction curves (see Fig. S2) displayed a steeper slope, hence, higher diversity for library Ca06, compared with the very similar curve progressions of libraries Ca07, Si06 and Si07. The high reciprocal Simpson index of 125 for library Ca06 indicated a diversity profile with relatively even distribution of the different OTU_S_, but also represented a marked contrast to the low index of < 50 for library Si06, which usually denotes a typical dominance profile (Stach *et al*., 2003). The Shannon–Weaver index suggested library Ca06 and Si06 as the most and least diverse libraries, respectively, although values did not greatly differ (Table 2).

**Table 2:** dotur-based richness and diversity estimates for the 16S-ITS rRNA clone libraries constructed from carbonate and silicate surface samples from December 2006 and August 2007

Sand type	Sampling time	Total clones	Total OTU_S_	Unique OTU_S_	Chao1 richness	Simpson index (1/D)	Shannon index (H)
Carbonate	Dec 2006	85	66	54	245 (145, 417)	125	4.1 (3.9, 4.3)
Carbonate	Aug 2007	72	48	34	110 (73, 202)	63	3.7 (3.5, 3.9)
Silicate	Dec 2006	53	38	32	203 (94, 596)	44	3.4 (3.2, 3.7)
Silicate	Aug 2007	73	51	39	133 (86, 247)	59	3.8 (3.5, 4.0)

The definition of OTU_S_ is based on a ≥ 98% identity. Chao1 richness and Shannon–Weaver indices are indicated with their 95% confidence interval in parentheses.

**Fig. 3:**
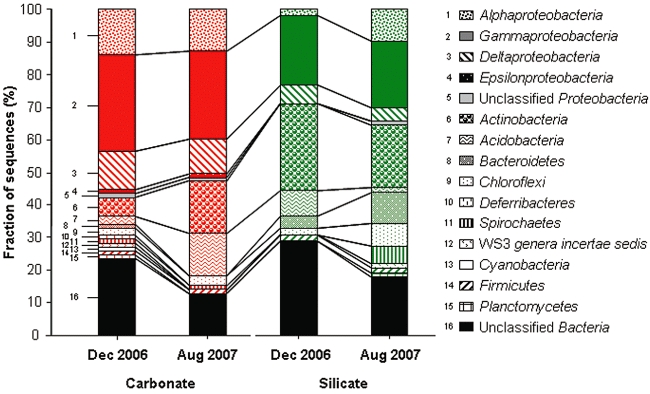
Frequencies of bacterial lineages detected in the 16S-ITS rRNA clone libraries constructed from carbonate and silicate surface samples from December 2006 and August 2007.

Noticeably, diversity patterns inferred from clone library analysis were very consistent with those inferred from ARISA (see Table S1A and B). Numerous OTU_S_ were affiliated with the phyla *Proteobacteria*, *Actinobacteria*, *Acidobacteria*, *Bacteroidetes*, *Chloroflexi* and *Spirochaetes* (Fig. 3; see also Table S2). The observed predominance of *Proteobacteria*-related sequences was well in concordance with previous studies on bacterial communities in permeable sands of two Hawaiian reefs (Sørensen *et al*., 2007; Rusch *et al*., 2009), the Great Barrier Reef (GBR; Uthicke and McGuire, 2007), as well as cold-water coral reefs (Yakimov *et al*., 2006). However, such pattern has also been reported from environments as contrasting as deltaic muds of Southeastern Papua New Guinea (Todorov *et al*., 2000), polar regions (Bowman and McCuaig, 2003) or the deep sea (Schauer *et al*., 2010). A complete description of taxonomic affiliations, including a comparison with other microbial diversity studies on permeable sands, is provided separately (see Supporting Information).

### Taxon-specific patterns of variation inferred from linking ARISA to taxonomy

A total of 74 (out of 423) OTU_A_ could be matched with 16S rRNA gene sequences and their respective taxonomic assignments at the bacterial class level (≥ 80% identity), resulting in an overall assignment success of 17.5% (see Table S2). Of those 74 identified OTU_A_, 67 OTU_A_* (asterisk indicates successful linking) were affiliated with a discrete taxon each, whereas 5 OTU_A_* corresponded to two distinct bacterial classes and 2 OTU_A_* corresponded to three phylogenetically unrelated clones.

The diversity dynamics obtained with the linked ARISA data were very similar to those inferred from the initial ARISA data (Mantel test *R* = 0.844, *P* < 0.001). It was therefore not surprising to observe that the main patterns of variation of OTU_A_*, when analysed by canonical RDA, were significantly related to sand type along the first ordination axis (representing 36.5% of the variance) and by season and sediment depth along the second axis (17.5% of the variance; see Fig. S3, Table S3A). While the variation in carbonate sands was positively correlated with two of the three seasons (i.e. August 2007 and February 2008) and with sediment depth, the exact opposite relationships were found in silicate sands, which once more illustrated the aforementioned sand type-specific variations in seasonal and vertical community response (see Table S3B). Furthermore, the relatively strong divergence among the different seasonal factor levels (see Fig. S3) re-emphasized the peculiarity of the winter (February 2008) samples in comparison to those collected during fall (December 2006) and summer (August 2007).

**Fig. 4:**
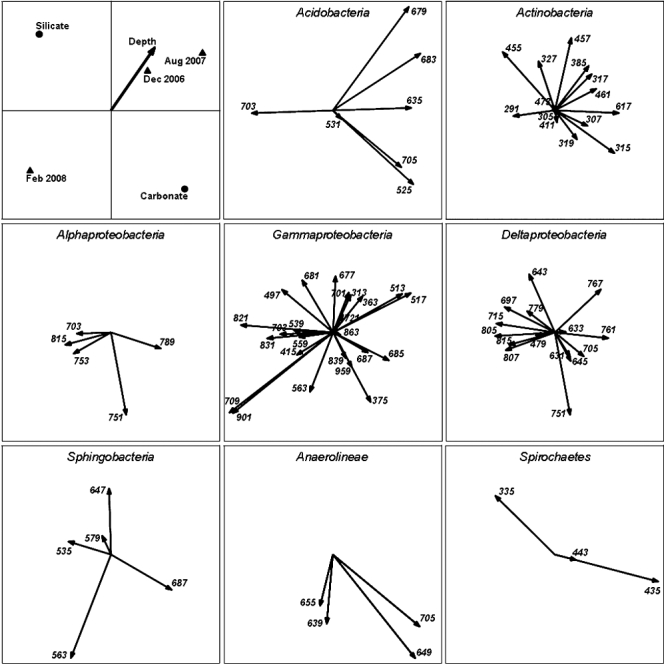
Relationships between sand type, season, sediment depth and changes in bacterial community structure, with focus on specific OTU_A_* shifts at the bacterial class level (≥ 80% identity). All biplots represent the same RDA ordination of linked ARISA data under direct constraint of explanatory factors, whereby the factor levels of sand type (circles) and season (triangles) were set as nominal variables, and those of sediment depth (vector) as continuous variables. Species vectors represent variation patterns of OTU_A_* that were linked to a 16S rRNA sequence of a given bacterial class, with numbers denoting the respective OTU_A_* (i.e. ARISA fragment) length in base pairs (for respective taxonomic assignments, see Table S2). Angles between vectors indicate the correlation between individual OTU_A_* distribution patterns, with collinear, opposite and orthogonal vectors, suggesting positive, negative and independent OTU_A_* covariation patterns respectively.

Individual RDA ordination plots depicting OTU_A_*–factor relationships for each of the most prominent different bacterial classes (Fig. 4) indicated various distribution patterns for members of the *Gammaproteobacteria*, *Deltaproteobacteria, Actinobacteria* and *Sphingobacteria*, which may reflect class-specific versatility in niche differentiation. In contrast, *Alphaproteobacteria* and *Spirochaetes* were mostly associated with the surface and middle layer, respectively, of both sand types in February 2008. *Rhodobacterales* (accounting for most of the *Alphaproteobacteria*-related OTU_A_*) usually alternate between chemoorganotrophic and phototrophic growth, which could explain their surface-specific occurrence. *Spirochaetes* are known as mainly anaerobic organisms, but OTU_A_* included in the analysis may as well comprise aerotolerant types. Furthermore, all *Anaerolineae* specifically grouped with the upper layer(s) of the carbonate samples only. How these strictly anaerobic organisms are able to thrive at the usually oxygen-rich sediment surface is unknown, but they may benefit from the presence of anoxic depressions on the carbonate grains as well as other metabolic strategies known from anaerobic lineages. *Acidobacteria* were mostly found in the middle and deep layer of December 2006 and August 2007, wherefore sampled members of this group (all *Acidobacteriales*) are assumed to favor suboxic or anoxic conditions.

As several ribosomal operons may exist within a bacterial cell (Klappenbach *et al*., 2000) and can greatly vary in length (Brown and Fuhrman, 2005), obtaining multiple OTU_A_* within a given bacterial lineage (e.g. *Gammaproteobacteria*, Fig. 4) cannot be directly equated with a high level of diversity (Brown *et al*., 2005). However, patterns of OTU_A_* were found to be very different within each bacterial class, suggesting that most of them may not originate from only few organisms, but rather reflect the high diversity also identified by clone library analysis (see Table S1A and B). This high diversity could be explained by the existence of various ecological responses within a given lineage to environmental conditions (as reconstructed by RDA; Fig. 4). Although the relationship between the presence of multiple rRNA operons from single organisms in response to resource availability was found to be insignificant in oligotrophic marine seawater (Brown and Fuhrman, 2005 and references therein), permeable reef sands, with their high input of different substrates and fast-changing biogeochemical conditions, may actually foster the concomitant development of several ecological strategies. Such possible diversification into ‘ecotypes’ with ecologically relevant physiological differences (Rocap *et al*., 2003) is assumed to support the relative fitness and overall resilience of a lineage in response to key environmental variables (Kitano, 2004).

In addition to RDA, regression analyses of individual OTU_A_* variations against each factor and respective factor levels were used to identify putative indicator OTU_A_* (see Table S4A–C). The selection of the 10 strongest OTU_A_*–factor relationships (with the highest and most significant R^2^) confirmed that mostly members of the *Gammaproteobacteria, Actinobacteria* and *Acidobacteria* were associated with overall type-, season- and sediment depth-related variations.

In conclusion, our results suggest that permeable biogenic carbonate and terrigenic silicate reef sands represent distinct and dynamic microbial habitats that harbour specific, comparably diverse bacterial communities over time and space. By offering locally contrasting environmental conditions, both sands contribute to an enhanced ecological structuring of bacterial diversity and potential functions within a single reef ecosystem. Many of the organisms identified in this study were also found in other reef or permeable sediments. It would therefore be highly worthwhile to determine whether molecular techniques offering higher taxonomic resolution levels and insights into functional differences could further advance our understanding of ecological patterns in reef-associated microbial communities.

## Experimental procedures

### Study site and sample collection

The study was conducted in a shallow fringing reef in the north-eastern Gulf of Aqaba, Red Sea, located within a marine reserve close to the Marine Science Station (29°27′N, 34°58′E; see also Wild *et al*., *2*005). During three field expeditions (December 2006, August 2007, February 2008, with average water temperatures of 23°C, 27°C and 21°C respectively), permeable reef sediments were sampled by SCUBA at two neighbouring reef sites, whereof the first site (2.5 m water depth) was covered by biogenic carbonate sands and the second (1.8 m water depth) by terrigenic silicate sands. Both sites were located within 1 m from the reef crest and about 150 m apart from each other. At each site, samples were collected in three different spots (approximately 50 cm apart) within a total patch area of 2 m^2^, using two scaled clipboards and sterile metal spoons. Each of the three spots was sampled in sediment depths of 0–2 cm (‘surface layer’), 2–6 cm (‘middle layer’) and 6–12 cm (‘deep layer’). Triplicates obtained for one depth horizon were directly transferred into a 15 ml tube, thereby producing a pooled sample for each layer. Between seasons, the same spots were re-sampled with an allowed error of only few centimetres from the original location, so as to limit sampling artefact in data collection. In February 2008, additional carbonate and silicate surface sands (0–2 cm) were collected outside the reef (‘out-reef’, 10 meters off the reef) at water depths of 4.1 m and 3.5 m, respectively, and in a distance of 10 m (silicate sands) and several hundred meters (carbonate sands) to both initial sampling sites (‘in-reef’). Within 1 h after collection, all samples were transported to the laboratory, homogenized with a sterile spatula, and transferred into 2 ml tubes. Aliquots for sediment characterization were processed directly, while those for DNA-based analyses (ARISA and 16S-ITS rRNA clone libraries) were frozen at −20°C until further use. Aliquots for microbial cell enumeration were fixed with 4% paraformaldehyde (PFA), incubated on a shaker at 4°C overnight, and washed twice with a sterile seawater-ethanol solution (1:1) prior to storage at −20°C.

### Sediment characterization

Carbonate content was measured by complexometric titration according to Muller (1967) and adapted by Rasheed and colleagues (2003b). Grain size and sorting coefficient were determined by fractional sieving based on Wentworth scaling (Wentworth, 1922).

The sedimentological properties of carbonate and silicate surface (0–2 cm) samples from February 2008 differed greatly between both sand types. While the biogenic carbonate sediments had an expectedly high CaCO_3_ content of 86.7%, the terrigenic silicate sediments consisted of only 19.3% CaCO_3_, with the remainder representing quartzous components (see also Rasheed *et al*., 2003b). Due to a grain size median of 553 µm and a sorting coefficient of 0.84, the carbonate sediments classified within the coarse sands (500–1000 µm; Wentworth, 1922) and exhibited only moderate sorting, indicating a relatively high level of heterogeneity due to the presence of many different grain fractions. The silicate sediments, on the contrary, appeared to be of smaller grain size (326 µm) and represented typical medium sands (250–500 µm median range). Their low sorting coefficient of 0.0016 is known to be characteristic for very well sorted, homogeneous sediments containing relatively few different grain fractions.

### Microbial cell enumeration

Enumeration of sand-associated microbial cells was performed with triplicate carbonate and silicate surface (0–2 cm) samples from February 2008. Following an optimized protocol for sandy sediments (Wild *et al*., 2006), microbial cells were first extracted from PFA-preserved samples by applying ultrasound in combination with acetic acid, and subsequently subjected to the acridine orange direct count (AODC) method. Microbial cells on filter wedges were counted in 25 randomly chosen fields. For the carbonate samples, obtained average and standard deviation of all counts were multiplied with the correction factor 1.87 (Wild *et al*., 2006) in order to account for the embedding of cells in the carbonate matrix.

### DNA extraction

From 0.5–1 g sediment sample, 3–5 replicates of total genomic DNA were extracted with the UltraClean Soil DNA Isolation Kit (MoBio, Carlsbad, CA, USA) following the manufacturer's instructions for maximum yield. Final elution of DNA was performed with 50–100 µl 1× TE buffer (Promega, Madison, WI, USA). Concentration of yielded DNA was determined using NanoDrop spectrophotometry (NanoDrop, Wilmington, DE, USA).

### ARISA fingerprinting

Bacterial ARISA (Fisher and Triplett, 1999) on replicated DNA extracts as well as subsequent data transformation and binning were carried out as described previously (Ramette, 2009) with slight modifications. The resulting response table (‘initial ARISA’ data), containing relative peak areas for all binned operational taxonomic units (OTU_A_), was used for multivariate analyses and for further coupling single OTU_A_ with their 16S rRNA sequence information.

### 16S-ITS rRNA clone library construction

For the construction of 16S-ITS rRNA clone libraries, carbonate and silicate surface samples with the highest OTU_A_ numbers were selected (December 2006 and August 2007 surface sample sets). PCR amplification of the 16S-ITS rRNA region was performed with bacterial primers 27f (5′-AGA GTT TGA TCM TGG CTC AG-3′; Lane, 1991) and ITSReub (5′-GCC AAG GCA TCC ACC-3′; Cardinale *et al*., 2004), yielding fragments of potential lengths between 1500 and 3000 bp length (see Supporting Information). Sequences have been deposited under GenBank accession numbers FR851476-FR851758 (partial 16S rRNA gene) and FR851759-FR851843 (ITS1).

### Taxonomic classification and diversity indices

For a total of 283 sequences (each with an average length of the 16S rRNA gene of 1050 bp), taxonomic affiliations were determined using the RDP Classifier and SeqMatch functions (Ribosomal Database Project II, Release 9.59; Cole *et al*., 2009). Further, these sequences were imported into the ARB software package (Ludwig *et al*., 2004) and aligned by applying the SILVA incremental aligner tool (SINA; Pruesse *et al*., 2007), including manual alignment correction.

Using an OTU definition of ≥ 98% identity for the sequence data set (referred to as OTU_s_), rarefaction curves, full-bias corrected richness estimators and diversity indices were computed using dotur (Schloss and Handelsman, 2005) after generating genetic distance matrices in ARB using the Jukes–Cantor correction. To determine whether differences in library composition were significant, the statistical tool ∫-LIBSHUFF was applied to genetic distance matrices, with significances assessed by Monte Carlo permutations and further corrected for multiple comparisons (Schloss *et al*., 2004). The statistical tool SONS (Schloss and Handelsman, 2006) was used on 16S rRNA sequences to calculate the shared Chao1 (shared richness), J_class_ (community overlap), and Theta_yc_ (community structure similarity) estimators.

### Coupling of ARISA to 16S rRNA sequence information

Taxonomic information was linked to OTU_A_ as described by Brown and colleagues (2005). For each sequenced clone, the length between (and including) the 27f and ITSReub primer sites was calculated and designated as the derived OTU_A_ length. Respective taxonomic information was inferred from the corresponding 16S rRNA gene portion, using an 80% bootstrap confidence support at the bacterial class level (RDP Classifier). All identified OTU_A_ (denoted OTU_A_*) were subsequently processed as a new table (‘linked ARISA’ data) for studying overall and individual patterns of variation.

### Statistical analyses

The initial ARISA data reflecting relative OTU_A_ abundance were used to calculate Bray–Curtis pairwise distances between samples, which were further visualized in a lower dimensional space by applying non-metric multidimensional scaling (NMDS) and cluster analysis. Analysis of similarity (anosim) was performed to test for significant differences between *a posteriori* sample groupings, and significance was corrected for multiple testing by the Bonferroni criterion. The relative importance of factors in explaining community variation was investigated by variation partitioning based on canonical redundancy analysis (RDA) of Hellinger-transformed data (Ramette and Tiedje, 2007). Single and combined fractions of variation were tested for significance by performing 999 Monte Carlo permutations. Furthermore, the initial ARISA data reflecting OTU_A_ presence–absence were used to compare mean OTU_A_ numbers by an overall KW test and subsequent pairwise Wilcoxon–Mann–Whitney tests.

Prior to analysing OTU_A_*-specific variation in community structure, the initial and linked ARISA data were tested for concordance in bacterial community variation by applying the Mantel test based on the corresponding Bray–Curtis distance matrices. Individual OTU_A_* responses to environmental effects were examined by RDA focusing on inter-species correlations at the bacterial class level (≥ 80% identity), while the response of discrete OTU_A_* and respective bacterial classes were monitored by single regression analyses as well as the Dufrene–Legendre indicator species analysis (Dufrene and Legendre, 1997). All statistical tests and graphics were performed in R v.2.9 (The R Project for Statistical Computing) using packages *stats*, *vegan*, *MASS*, *labdsv*, *mgcv*, and CANOCO for Windows v4.5 (terBraak and Smilauer, 2002).

## Supporting information

**Fig. S1.** ARISA-based OTU_A_ partitioning and overlap between carbonate and silicate sands at different seasons and sediment depths.

**Fig. S2.** dotur-based rarefaction curves for the 16S-ITS rRNA clone libraries constructed from carbonate and silicate surface (0–2 cm) samples from December 2006 and August 2007, using the furthest neighbour assignment algorithm. Error bars represent the upper and lower bound of the 95% confidence interval.

**Fig. S3.** Relationship between factors sand type, season and sediment depth, and changes in bacterial community structure, with focus on specific OTU_A_^*^ shifts at the bacterial class level (≥ 80% identity). The biplot represents a RDA ordination of linked ARISA data under direct constraint of explanatory factors, whereby the factor levels of sand type (circles) and season (triangles) were set as nominal variables, and those of sediment depth (vector) as continuous variables. Species vectors represent all 76 OTU_A_^*^ that were linked to a 16S rRNA sequence of a given bacterial class, with numbers indicating the respective OTU_A_^*^ (i.e. ARISA fragment) length in base pairs. Angles between vectors indicate the correlation between individual OTU_A_^*^ distribution patterns, with collinear, opposite and orthogonal vectors suggesting positive, negative and independent OTU_A_^*^ covariation patterns respectively. For respective taxonomic assignments, see Table S2.

**Table S1A.** ∫-LIBSHUFF-based pairwise comparisons of community difference for the 16S-ITS rRNA clone libraries constructed from carbonate and silicate surface (0.2 cm) samples from December 2006 and August 2007.

**Table S1B.** SONS-based pairwise estimates of shared richness (Shared Chao1), community overlap (J_class_) and community structure similarity (Theta_YC_) for the bacterial 16S-ITS rRNA clone libraries constructed from carbonate and silicate surface (0–2 cm) samples from December 2006 and August 2007.

**Table S2.** Compilation of ARISA-derived OTU_A_^*^ that were successfully linked to 16S rRNA taxonomy, based on RDP Classifier and SeqMatch analyses.

**Table S3A.** RDA-derived correlations between single factor levels and the two main RDA axes.

**Table S3B.** RDA-derived correlations among single explanatory factors and factor levels.

**Table S4A.** Regression analyses of single bacterial OTU_A_^*^ against factor sand type (and single factor levels) by partialling out the effects of season and sediment depth.

**Table S4B.** Regression analyses of single bacterial OTU_A_^*^ against factor season (and single factor levels) by partialling out the effects of sand type and sediment depth.

**Table S4C.** Regression analyses of single bacterial OTU_A_^*^ against factor sediment depth (and single factor levels) by partialling out the effects of sand type and season.
